# Full-optical photoacoustic imaging using speckle analysis and resolution enhancement by orthogonal pump patterns projection

**DOI:** 10.1038/s41598-023-45490-9

**Published:** 2023-10-23

**Authors:** Viktor Vorobev, David Weidmann, Sergey Agdarov, Yafim Beiderman, Nadav Shabairou, Matan Benyamin, Florian Klämpfl, Michael Schmidt, Dmitry Gorin, Zeev Zalevsky

**Affiliations:** 1 Center for Photonic Science and Engineering, Skolkovo Innovation Center, Skolkovo Institute of Science and Technology, Moscow, Russia 143026; 2 Faculty of Engineering, Bar-Ilan University, 52900 Ramat-Gan, Israel; 3 Lehrstuhl für Photonische Technologien, Friedrich-Alexander-Universität Erlangen-Nürnberg, 91052 Erlangen, Germany

**Keywords:** Photoacoustics, Imaging and sensing

## Abstract

This paper presents an approach for achieving full optical photoacoustic imaging with enhanced resolution utilizing speckle pattern analysis. The proposed technique involves projecting patterns derived from binary masks corresponding to orthogonal functions onto the target to elicit a photoacoustic signal. The resulting signal is then recorded using a high-speed camera and analyzed using correlation analysis of the speckle motion. Our results demonstrate the feasibility of this optical approach to achieve imaging with enhanced resolution without the need for physical contact with the target, opening up new possibilities for non-invasive medical imaging and other applications.

## Introduction

Photoacoustic (optoacoustic) imaging (PAI) is a non-invasive visualization modality that recently has gained a significant attention in the fields of biophotonics and biomedical optics^1–24^. The technique is based on the photoacoustic (optoacoustic) effect, which is the generation of acoustic waves in a material after it absorbs light and transfers energy to thermoelastic expansion. In common form of PAI, a short-pulsed pump laser used to illuminate the material. Subsequent acoustic waves detected either acoustically, using an ultrasound transducer, or optically. The recorded information used to generate tissue images. The amplitude of the photoacoustic effect is dependent on the optical absorption coefficient of the material. Because different tissue types usually have different absorption coefficients, the PA effect can be used to distinguish between them^7^.

PAI is a hybrid modality that combines photonics and ultrasound detection. The optical component of PAI enables one to utilize spectral specificity to distinguishes between different absorbers. Considering Abbe’s law, the use of optical wavelengths allows to achieve a reasonably high spatial resolution. However, the detection is limited by the shallow depth of light penetration^4–6^. The ultrasound component of PAI serves as a complementary modality that enables the collection of information from deeper lying tissues as compared to optical techniques.

PAI leverages the benefits of each technique, enabling the acquisition of functional information with high-resolution and at depths that are challenging to access using conventional optical imaging techniques or ultrasound imaging alone^4^.

Overall, PAI is an important imaging modality with a wide range of potential applications in biomedical imaging. Its ability to provide high-resolution and functional information about tissue makes it a promising tool for the diagnosis and monitoring of various diseases, including cancer, cardiovascular diseases, bowel disease, and neurological disorders^8,10,20–22,24^.

Previously, the concept of space bandwidth adaptation was developed as a new methodology to analyze and understand optical imaging systems and to adjust their resolution by performing conversion of spatial degrees of freedom^25–27^. Large numbers of super resolving concepts were introduced to overcome diffraction-related limitation of resolution by performing time multiplexing^28,29^, field of view multiplexing^30,31^, wavelengths multiplexing^32,33^, coherence states multiplexing^34,35^, polarization multiplexing^36,37^, gray level encoding^38^ and more and also to over-come the geometric limit of resolution^39^.

In this work we aimed to combine PAI with spatial resolution enhancement techniques.

## Theory

PA signals require a high temporal sampling rate and resolution. The problem is that hardware-wise, high temporal sampling rates are opposed to high spatial resolution. Previously, in Ref^40^. it was shown that it is possible to construct detectors with high temporal resolutions of several MHz. But, they had only few pixels and thus they produced signals of low spatial resolution.

In this work we combined time-multiplexing and resolution enhancement concepts in order to use the high temporal and low spatial resolution detectors to obtain high temporally and spatially resolved PA sensing. The idea is to project on the imaged object a set of (time changing) a-priori known encoding patterns in order to decode the high spatial resolution. Such encoding patterns can be, e.g., projected a-priori known speckle patterns (random encoding patterns). The basics of the proposed approach was investigated in Ref.^41^, but never implemented with high resolution temporal detectors, as we did in our current study.

Mathematical proof of such a concept can be shown as follows. We will denote by Pn(x) the set of projected patterns, that acts as an orthogonal basis for compressed sensing decomposition. We assume we have N such patterns (the number of the functions in the decomposition basis). We will denote by s(x) the object to be imaged.

The readout value we get per projected pattern of Pn(x) is:

1 $$r\left[n\right]=\int s\left(x\right){P}_{n}\left(x\right)dx$$

The reconstruction of the object from the set of readouts is:

2 $$\widehat{s}\left(x\right)=\sum_{n}r\left[n\right]{{P}_{n}}^{*}\left(x\right)$$

Let us now prove it. We start by substituting Eq. (1) into Eq. (2):

3 $$\widehat{s}\left(x\right)=\sum_{n}\int s({x}^{\mathrm{^{\prime}}}){{{P}_{n}\left(x\mathrm{^{\prime}}\right)P}_{n}}^{*}\left(x\right)dx\mathrm{^{\prime}}=\int s({x}^{\mathrm{^{\prime}}})\left(\sum_{n}{{{P}_{n}\left(x\mathrm{^{\prime}}\right)P}_{n}}^{*}\left(x\right)\right)d{x}^{\mathrm{^{\prime}}}$$

Due to the orthogonality property of the projected complete basis, one has:

4 $$\sum_{n}{{{P}_{n}\left({x}{\prime}\right)P}_{n}}^{*}\left(x\right)\approx \delta \left(x-{x}^{\mathrm{^{\prime}}}\right)$$

By substituting it in Eq. 3 one obtains:

5 $$\widehat{s}\left(x\right)=\int s\left({x}^{\mathrm{^{\prime}}}\right)\delta \left(x-{x}^{\mathrm{^{\prime}}}\right)d{x}^{\mathrm{^{\prime}}}=s\left(x\right)$$

Thus, the reconstruction of the spatial pattern is equal to the original spatial distribution. This was done by collecting the information via only a single point detector. By such means it is possible to accomplish enhanced spatial resolution.

We chose Walsh functions as the set of the projected patterns *P*_*n*_(*x*), with one notable modification. The Original Walsh functions are constructed in the binary base of -1 and 1. Experimentally, measurements were done with binary masks, so projected patterns were constructed in the binary base of 0 and 1. Reconstruction in this case is done in the same way, but with the following modifications.

Next, we will operate in the basis where Walsh functions are constructed in the base of -1/2 and 1/2. We will denote by ${P}_{n}^{ort}\left(x\right)$ the set of orthogonal base Walsh functions and the set of non-orthogonal projected patterns based on Walsh functions by ${P}_{n}^{non ort}(x)$. Thus, one can see that ${P}_{n}^{ort}\left(x\right)= {P}_{n}^{non ort}\left(x\right)-\frac{1}{2}E$, where E is a matrix of ones.

The readout value per projected pattern of ${P}_{n}^{ort}(x)$ is written in Eq. 1. The readout value per projected pattern of ${P}_{n}^{nonort}\left(x\right)$ is:

$${r}^{non ort}[n]=\int s\left(x\right){P}_{n}^{non ort}\left(x\right)dx =$$

6 $$=\int s\left(x\right){P}_{n}^{ort}\left(x\right)dx+\frac{1}{2}E\int s\left(x\right)dx={r}^{ort}\left[n\right]+ \frac{1}{2}E\int s\left(x\right)dx$$

Now, the reconstruction of the object should be done as follows:

7 $$\begin{aligned} \hat{s}\left( x \right) = & \mathop \sum \limits_{n} r^{ort} \left[ n \right]P_{n}^{ort} \left( x \right) = \mathop \sum \limits_{n} \left( {r^{{non{ }ort}} \left[ n \right] - { }\frac{1}{2}E\smallint s\left( x \right)dx} \right)P_{n}^{ort} \left( x \right) \\ = & { }\mathop \sum \limits_{n} r^{{non{ }ort}} \left[ n \right]P_{n}^{ort} \left( x \right) - { }\mathop \sum \limits_{n} \frac{1}{2}E\smallint s\left( x \right)dxP_{n}^{ort} \left( x \right){ } \\ = & { }\mathop \sum \limits_{n} r^{{non{ }ort}} \left[ n \right]P_{n}^{ort} \left( x \right) - { }\frac{1}{2}r^{{non{ }ort}} \left[ 0 \right]\mathop \sum \limits_{n} P_{n}^{ort} \left( x \right){ } \\ \end{aligned}$$

where we took advantage of the fact that measurements using the fully transparent Walsh mask located in the 1st row, 1st column and denoted here as n = 0, 0th mask can be expressed as:

8 $${r}^{non ort}\left[0\right]= E\int s\left(x\right)dx=\int s\left(x\right)Edx= \int s\left(x\right){P}_{0}^{non ort}\left(x\right)dx$$

A transformation of this kind, i.e. Hadamard-Walsh transformation, is closely related to the Fourier transformation and, as with the Fourier transformation, in this case we can obtain a reconstructed image of the desired object in the “frequency” domain.

We sense the PA signals optically, by analyzing the temporal changes in the secondary speckle patterns^42^, as well as acoustically, by analyzing voltage changes on transducer output over time.

## Methods

**Figure 1:**
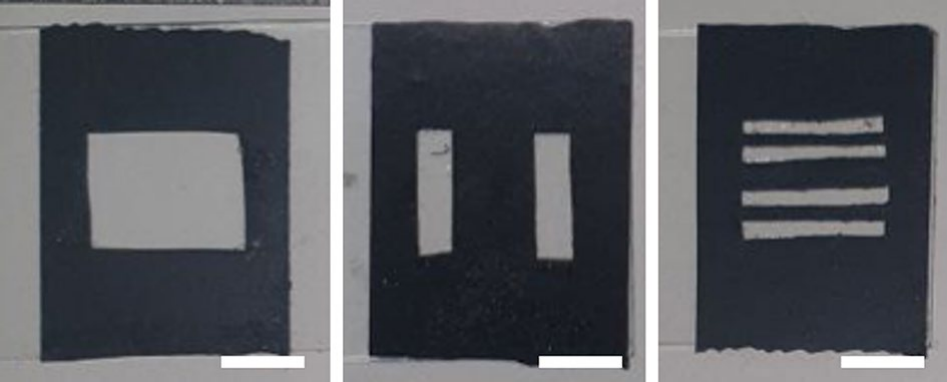
Binary masks are produced for measurements of different targets, scalebars − 1 cm.

Binary masks were constructed by applying opaque black tape onto a glass slide in a specific pattern, as illustrated in Fig. 1. The black tape was placed in such a way as to match the desired orthogonal function. Then, the masks were positioned in the path of the light beam to produce a spatially modulated projected pump beam. The dimensions of all masks and targets were equal and chosen to be consistent with the pump laser beam diameter.

For the optical part of this work, measurements were performed using a Fastcam Nova S12 (Photron, Japan) camera.

Recordings were taken with a temporal resolution of 100,000 fps and a camera resolution of 128 × 48 pixels, thus acting as a multi-point detector collecting information from 6144 pixels, simultaneously. Furthermore, a long pass filter was placed in front of the camera to block pump pulse laser radiation.

We used the Nd:YAG LPY-700 laser (Litron Lasers, Eng-land), which has a repetition rate of 10 Hz, a pulse duration of 6–9 ns, a wavelength of 532 nm and a nominal maximal energy output − of 600 mJ. The laser beam was collimated and its diameter was approximately 2 cm.

A probe laser was used to generate speckle images and to read the PA signal by sensing through the speckle patterns variations, the generated acoustic vibrations. Speckle patterns lateral motion was recorded by the camera. Thus, the PA signal’s acoustic vibrations were captured optically and used to measure the PA signal. For the probe laser we used the Helium–Neon CW laser HNL050R (Thorlabs, USA), with an emission wavelength of 633 nm. As a target, we arranged the black tape in patterns representing the binary masks on two different surfaces. The pattern was on glass for transducer measurements and on thin white tissue for speckle measurements. The transducer PA measurement was done as a reference, to compare it to the optically extracted PA results.

**Figure 2:**
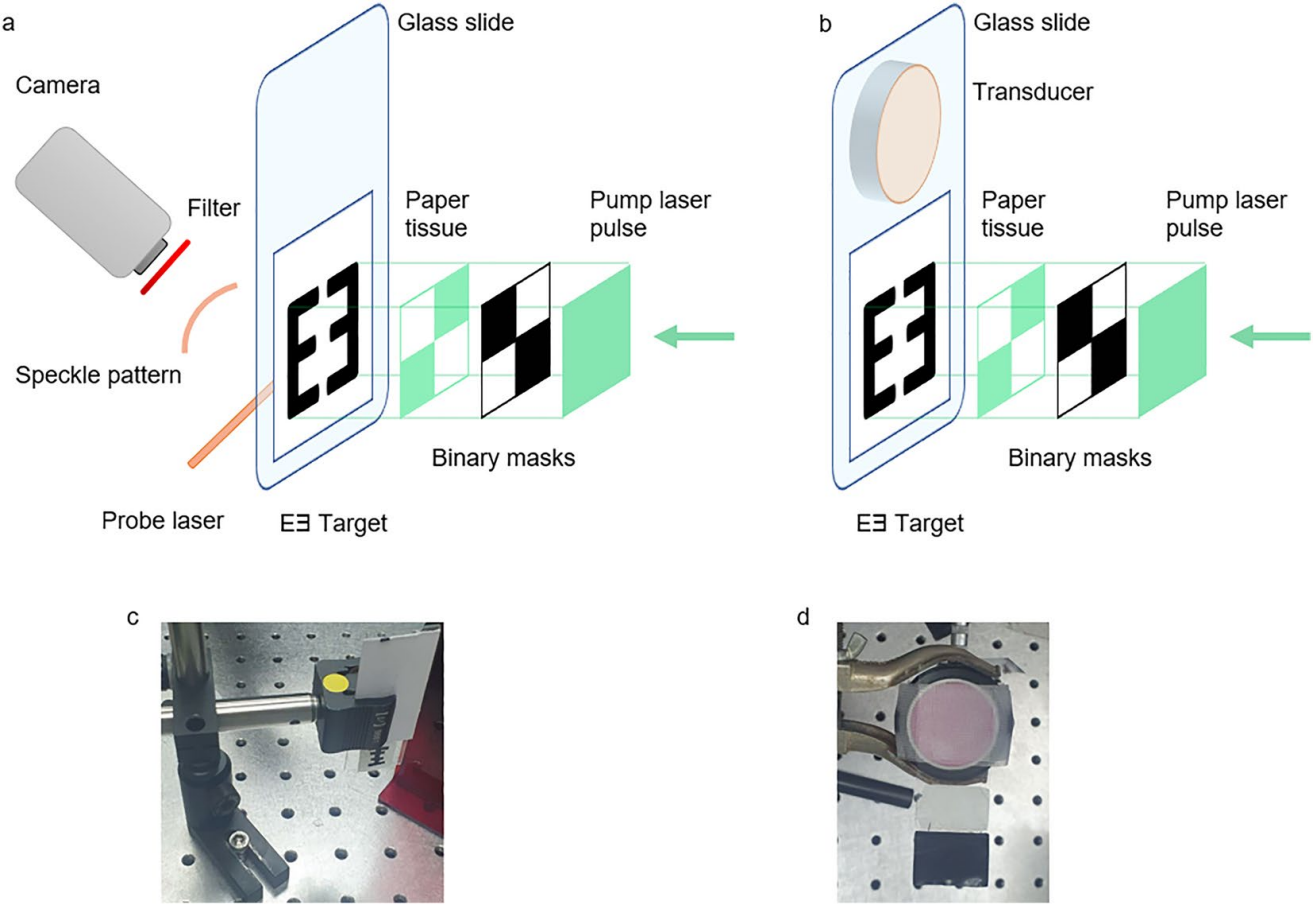
Experimental configurations. (**a**). Experimental setup for optical measurements with the camera, (**b**). acoustic measurements with transducer. The distance between binary mask and paper tissue was 30 cm. (**c**). Holder for PA targets used for the optical measurements, (**d**) holder for a transducer.

In the acoustic measurements part, we used an ultrasonic transducer CN4R-24 (Panametrics-NDTTM, USA), 4 MHz—central frequency, 24 mm. Since the transducer was a single-point detector, there was only one pixel of information. The oscilloscope was InfiniiVision DSO5014A (Agilent Technologies, USA) and the photodiode used to trigger oscilloscope measurements was DET10A (Thorlabs, USA). Schematics of the experimental setups used for the data collection are presented in Fig. 2.

For the optical part, a laser beam, spatially modulated by binary masks, was directed onto a black-tape target laid out in a specific pattern. The tape was laid out on white tissue so that the tape and tissue would oscillate, due to the PA effect, with an amplitude larger than that of a glass slide. It is clear that the amplitude of this oscillation correlates directly with the strength of the PA effect. For the optical part, higher amplitudes provide more noticeable movement of the speckle pattern. The speckle pattern was created by scattering the probe laser light from the central point of the target. For the speckle recording, the speckle pattern was positioned in the field of view of the camera and the camera was slightly defocused to meet requirements for use of the speckle patterns correlation analysis, as described in Ref.^43^.

For the ultrasound (US) measurements, performed as a reference, the PA signal was recorded indirectly by illuminating one side of the glass slide, where target was located, and measuring the US waves at the other. The black tape was taped directly onto the bottom part of the glass slide, and the glass slide was taped to the top part of the transducer, such that only the black tape was illuminated. This was done to isolate the PA effect and so that the laser light would not hit the transducer detector. Thus, the US waves were transmitted from the black tape to the transducer through the glass slide, which also allowed for low damping of the US waves.

Note that acoustical and optical systems are different and thus the consistency between signals was ensured by use of the same pump laser with the fixed parameters, same material (black tape) and target image for both approaches.

## Results

We demonstrate the feasibility of the proposed approach in the case of low spatial resolution for a 4 × 2 binary target. First, the Walsh transformed image of a given target was computed. The number of unique elements present in the Walsh transformation determined the number of masks necessary for the reconstruction process. This was because, if any two elements in the transformed target were identical, then only data obtained from a single mask that corresponded to that particular element had to be employed in the calculations.

**Figure 3:**
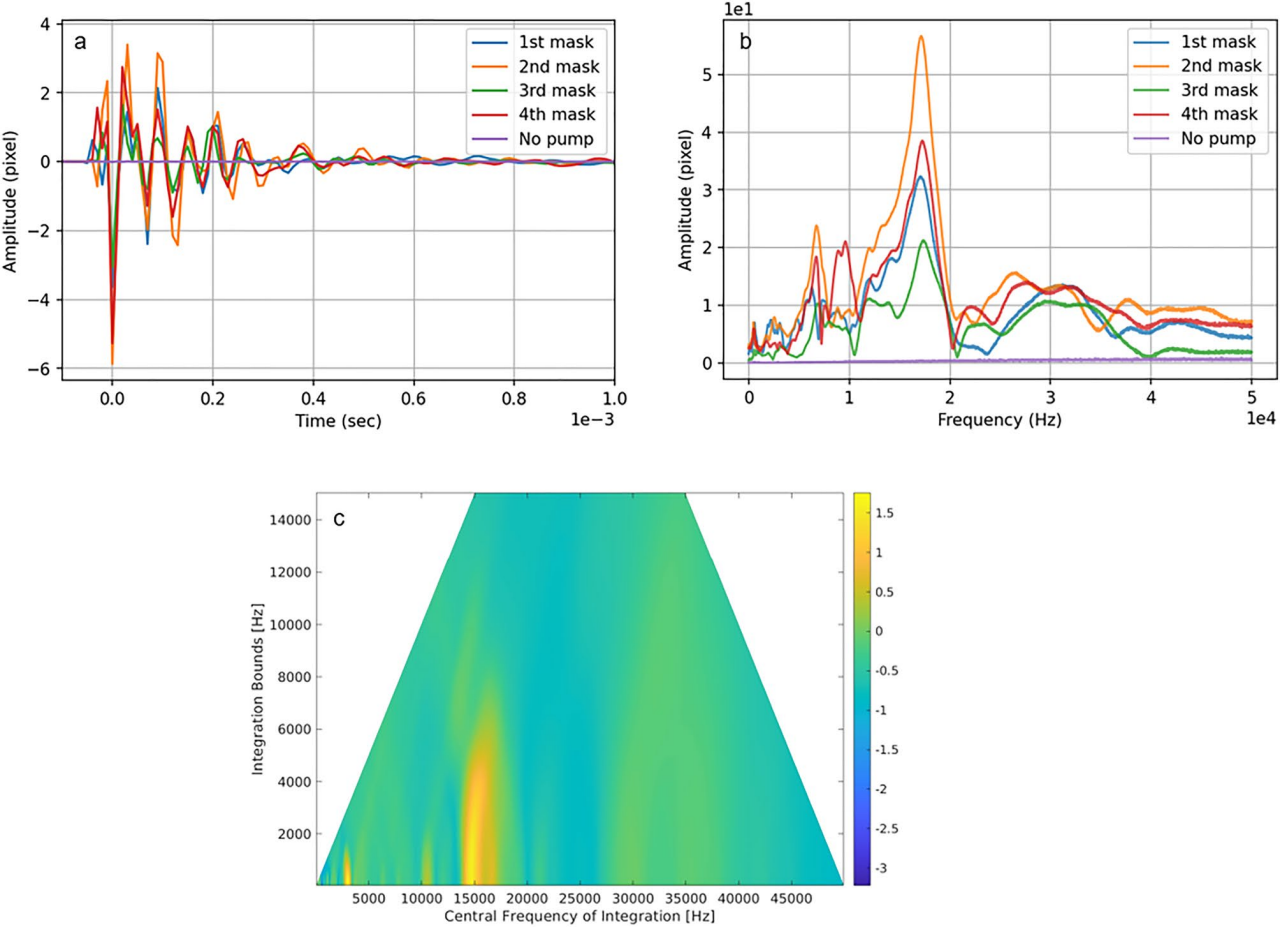
Experimental results. (**a**) Temporal signal for the speckle correlation peak movement along the Y axis, (**b**) frequency of the speckle correlation peak movement along Y axis, (**c**) difference between the means of the signal from the black tape and the tissue divided by the standard deviation of the signal from the tape, color bar shown in log scale.

The optical measurements-based reconstruction was performed as follows: A video recording captured the motion of the speckle pattern that arose from scattering a laser probe on white tissue. The resulting video was analyzed using the correlation approach, as explained in Ref.^43^, to extract the time-dependent correlation peak as the speckle moved along both the X and Y axes. The Fourier transformed signal in the frequency domain was then analyzed (Fig. 3, only Y axis movement depicted).

The computation of the readout signal involved integration over a specific range of frequencies, determined by central frequency and band. For each central frequency and band in the frequency domain, a reconstruction was done according to the method described in the introduction section. Then, two average values were calculated over two sets of pixels. The first set comprised of those pixels in which the PA signal is expected, and the second comprised of those pixels where the signal is not expected. Next, the difference between the average values of the first and second groups of pixels is divided by the standard deviation of the first group to obtain a ratio. These calculations are made for each center frequency and each band of integration (Fig. 3c). For the reconstruction shown in the results section, the maximum value of all calculated ratios was used. The logic behind this approach is that optimal target visualization is achieved when the contrast between the first and second groups, as well as uniformity of signal distribution among the first group, are as high as possible.

**Figure 4:**
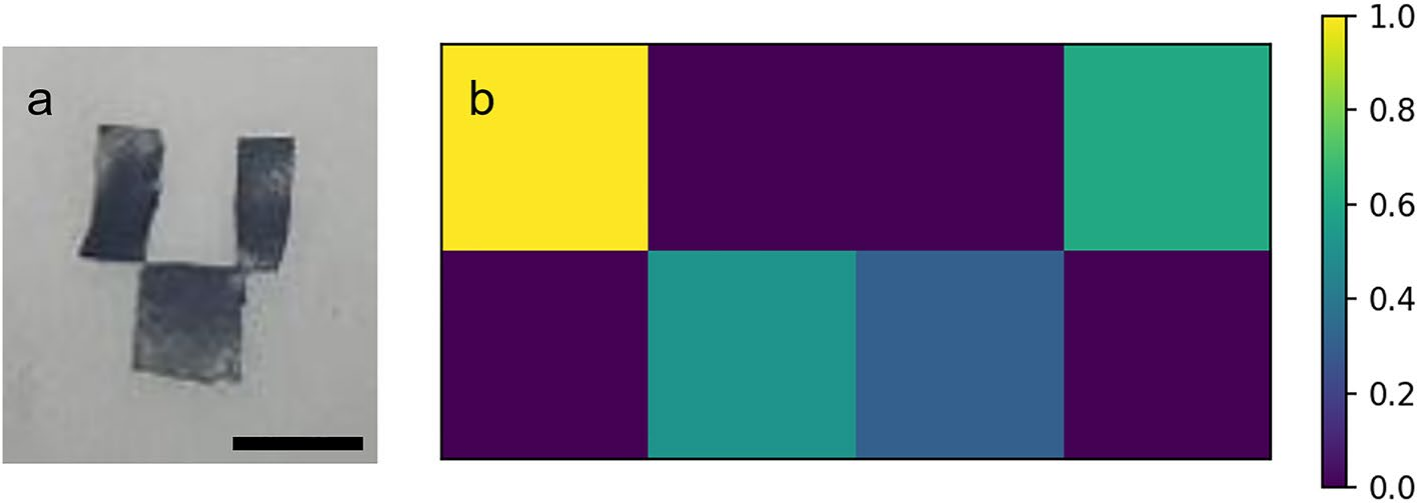
Imaging. (**a**) Target image, scalebar − 1 cm, (**b**) reconstructed image.

Thus, it is possible to identify the frequency band that offers the optimal contrast between the anticipated locations containing PA signals (black tape) and locations where they are not expected (tissue paper). The values obtained from calculation performed in the frequency domain were then used to generate the reconstructed image of the target. By applying the reconstruction technique outlined in the Theory section, the image depicted in Fig. 4 was obtained.

**Figure 5:**
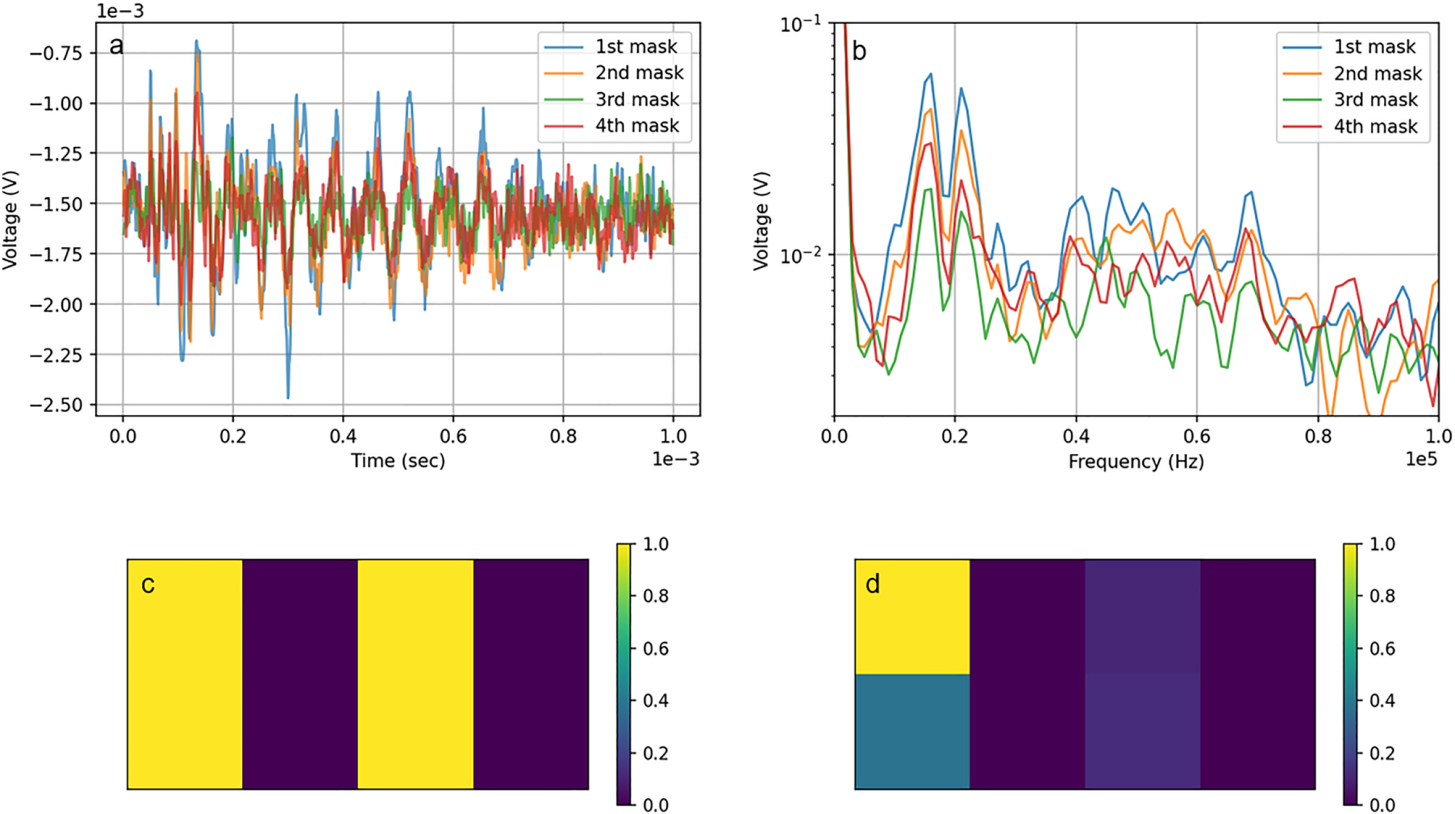
Experimenting. (**a**) Voltage changes on the transducer in the time domain, (**b**) voltage signal of transducer in the frequency domain, (**c**) digital image of the target, (**d**) reconstructed image of the target.

In the case of acoustic measurements, we recorded vibrations of the glass that were caused by the PA effect. Temporal changes of voltage within the transducer were recorded and then analyzed in the frequency domain (Fig. 5). The reconstruction process in this case was the same as in the case of the speckle measurements.

**Figure 6:**
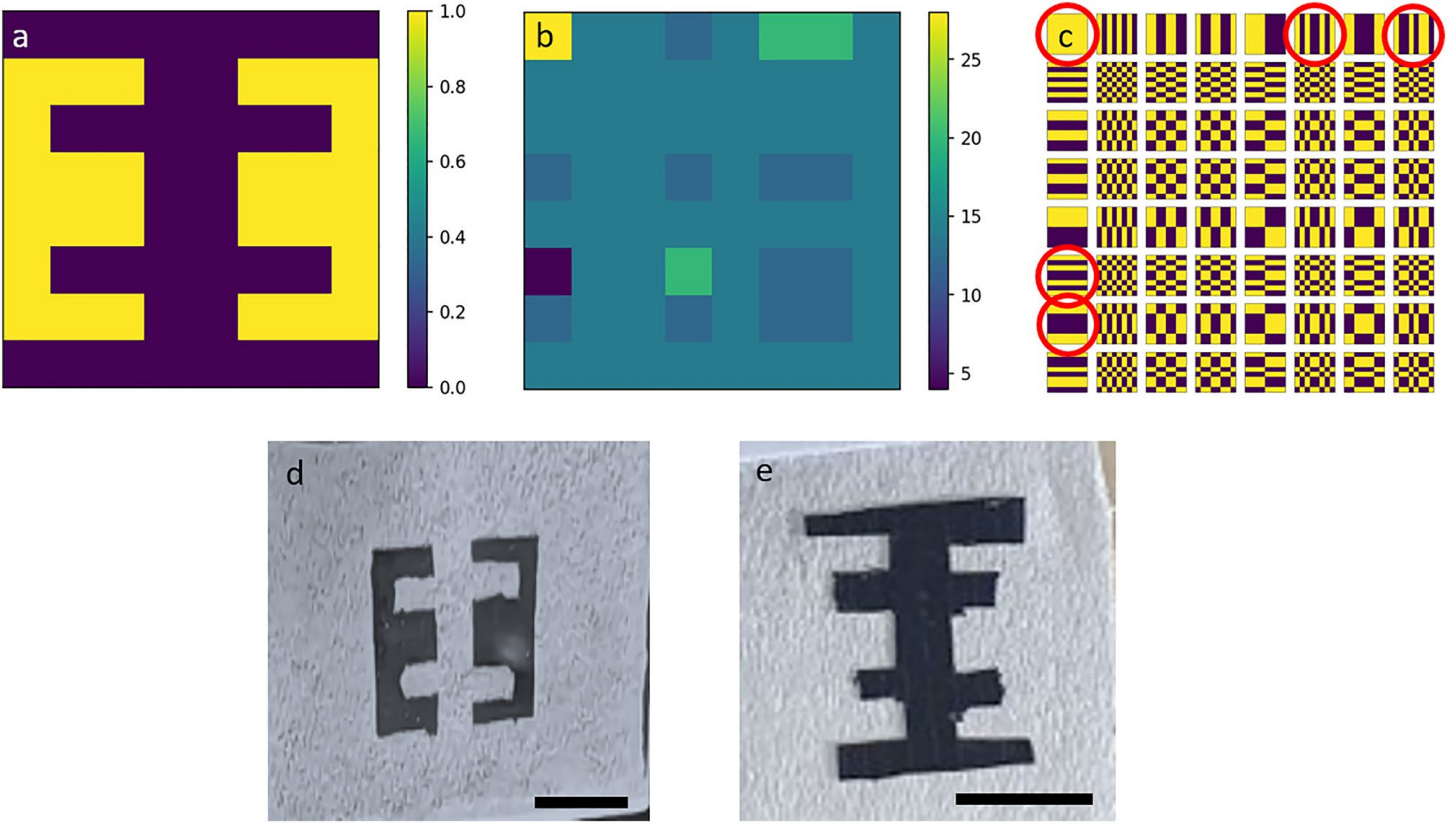
Super resolving experimenting. (**a**) “EƎ” target, (**b**) transformed image of the “EƎ” target, (**c**) complete set of orthogonal Walsh matrices for the 8 × 8 pixels case with 5 masks distinguished by red circles that corresponds to the 5 unique elements in the transformed image, (**d**) real “EƎ” target used for the measurements and (**e**). Inverted “EƎ” target, scalebars − 1 cm.

Finally, to prove that reconstruction at a higher spatial resolution is also possible, we performed reconstruction for a target having 8 × 8 pixels, an “EƎ” shape target. The results are shown in Fig. 6.

For this target, the number of unique elements is 5, which means that, theoretically, measurements of only 5 binary masks highlighted by red circles in Fig. 6, are needed. The target was measured using both optical and acoustic approaches. In the optical approach, measurements were conducted on both the target and its inverted form. In the acoustic approach, measurements were conducted only on the inverted target, since the signal from the standard “EƎ” target was low and signal to noise ratio was also low. This could be explained by the comparison between theoretical redout values (Eq. 1) for both targets when same patterns are projected. In case of standard “EƎ” target these values are: r[1, 1] = 28, r[1, 6] = 20, r[1, 8] = 14, r[1, 6] = 4, r[1, 7] = 12. On the other hand in case of inverted “EƎ” target these values are: r[1, 1] = 36, r[1, 6] = 12, r[1, 8] = 18, r[1, 6] = 28, r[1, 7] = 20. Thus, inverted “EƎ” target in general provides higher signal during measurements, yielding higher SNR for the reconstructed image.

**Figure 7:**
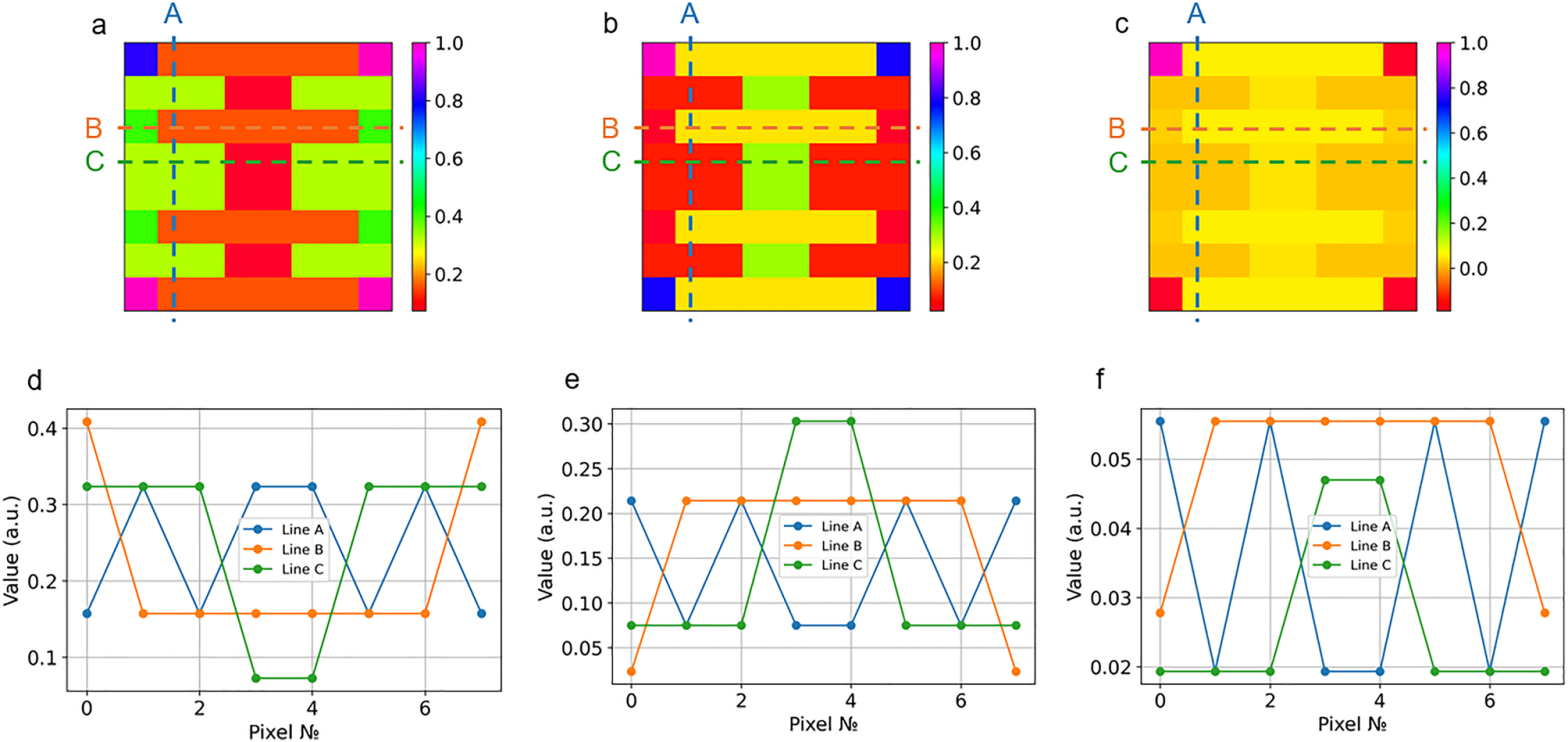
Experimental results. (**a**) Reconstructed image of “EƎ” target in the case of speckle measurements, (**b**) reconstructed image of the inverted “EƎ” target in the case of speckle measurements and (**c**) reconstructed image of the inverted “EƎ” target in the case of acoustic measurements. (**d**), (**e**), (**f**) The signal along the lines A, B, C of the reconstructed images (**a**), (**b**) and (**c**), respectively.

The measurements obtained were processed using the same methods as described in the 4 × 2 pixels case, and the reconstructed image is presented in Fig. 7. Note that this is an experimental example of the resolution enhanced PA sensing since by having only 5 readouts we aim to reconstruct an 8 × 8 pixels object. A reduced number of measurements was possible to use, because we already knew the image we were looking for. In the case of measuring a previously unknown image, measurements must be made on a full set of orthogonal pump patterns.

The appearance of artifacts in the four corner pixels of the reconstructed image was anticipated and can be attributed to the use of only five masks in the measurement process, rather than the full set of 64 masks, resulting in an increased susceptibility to measurement errors (see SI for details). Despite this, the desired “EƎ” target was distinguishable in all images. Notably, in the optical approach, the values along lines A, B, and C for both the normal and inverted targets were consistent with the expected values and exhibited an inverted relationship with each other. Based on the obtained results, it is evident that the proposed methodology not only enables non-contact PAI but also yields images exhibiting approximately twofold greater contrast when compared to measurements acquired using contact transducer, as depicted by lines B and C in Fig. 7b,c. In our experimental investigations, the optical approach demonstrated greater sensitivity in comparison to contact-based measurements. Sensitivity for optical approach was especially pronounced in case of the standard "EƎ" target, for which the contact acoustic approach showed insufficient sensitivity.

## Discussion

In this study, we explored the potential implementation of time-multiplexing resolution enhancement techniques for improving the quality of PA imaging. Our results demonstrate that, by using optical speckle correlation analysis approaches for probing, it is possible to reconstruct the photoacoustic target image with satisfactory accuracy. Here we successfully showed resolution enhancement for PA targets using both single-point (transducer) and multipoint (camera) detectors. This was achieved by projecting orthogonal base Walsh functions onto the target to induce a PA response. The response was detected either by a transducer or by recording the movement of the speckle patterns by a high-speed camera. The collected PA signals were then analyzed in the frequency domain and utilized for image reconstruction. Our findings demonstrate a high correlation between the reconstructed images and the original targets. Overall, this study demonstrates the potential of resolution enhancement techniques to improve the diagnostic capabilities of optoacoustic imaging. Next steps for the development of this approach involve use of a spatial light modulator for more precise patterns projection and automating the process. Future work could focus on further developing and refining these techniques to improve their accuracy and applicability for a wide range of clinical and research related applications.

Note that the camera affects the quality reconstruction by its limited spatial (pixels) and temporal resolution (its frame rate). In this study the speckles were large and were sampled by several pixels of the camera and thus the spatial resolution did not cause reconstruction error. Unlike in the case of using a transducer that has a single pixel. The frame rate of the camera although was quite high affected the quality of reconstruction. Therefore, in the overall the camera affects the reconstruction and adds reconstruction errors. Such errors were calculated by correlating the reconstructed image with the original one. This is presented in Figure S6. Possible ways to overcome the temporal limitations imposed by the camera’s frame rate will be addressed in our future publications.

Also note that the main purpose of this work was to preliminary demonstrate the novel technique itself and provide a basis for its future developments. As this approach is novel, to the best of our knowledge, it was crucial to perform first measurements on test, samples in order to provide well-founded demonstration of such technique and confirm that it is indeed provides us with reasonable results. The outcome of our work proves that it is promising to investigate this approach further and more thoroughly as we indeed intend to perform in our future work in this direction.

### Supplementary Information

Supplementary Information.

## Data Availability

Upon reasonable request, contact VV.

## Code availability

Upon reasonable request, contact VV.
