# Supplementary material for: Full-optical photoacoustic imaging using speckle analysis and resolution enhancement by orthogonal pump patterns projection
Source: Sci Rep. 2023 Oct 23;13:18081. doi: 10.1038/s41598-023-45490-9 (PMC10593755; doi:10.1038/s41598-023-45490-9)
Supplement: Supplementary file 1 — Supplementary Information. [file 41598_2023_45490_MOESM1_ESM.docx]

*-Supporting Information-*

Full-optical Photoacoustic Imaging Using Speckle Analysis and Resolution Enhancement by Orthogonal Pump Patterns Projection

Viktor Vorobev^1, ‡^, David Weidmann^2, ‡^, Sergey Agdarov^2^, Yafim Beiderman^2^, Nadav Shabairou^2^, Matan Benyamin^2^, Florian Klämpfl^3^, Michael Schmidt^3^, Dmitry Gorin^1^ and Zeev Zalevsky^2,3^*

^1^ Center for Photonic Science and Engineering, Skolkovo Institute of Science and Technology, Skolkovo Innovation Center, Moscow 143026, Russia

^2^ Faculty of Engineering, Bar-Ilan University, Ramat-Gan 52900, Israel

^3^ Friedrich-Alexander-Universität Erlangen-Nürnberg, Lehrstuhl für Photonische Technologien, Konrad-Zuse-Straße 3/5, 91052 Erlangen

KEYWORDS: Photoacoustic imaging, Speckle interferometry, Patterns projection, Super-Resolution.


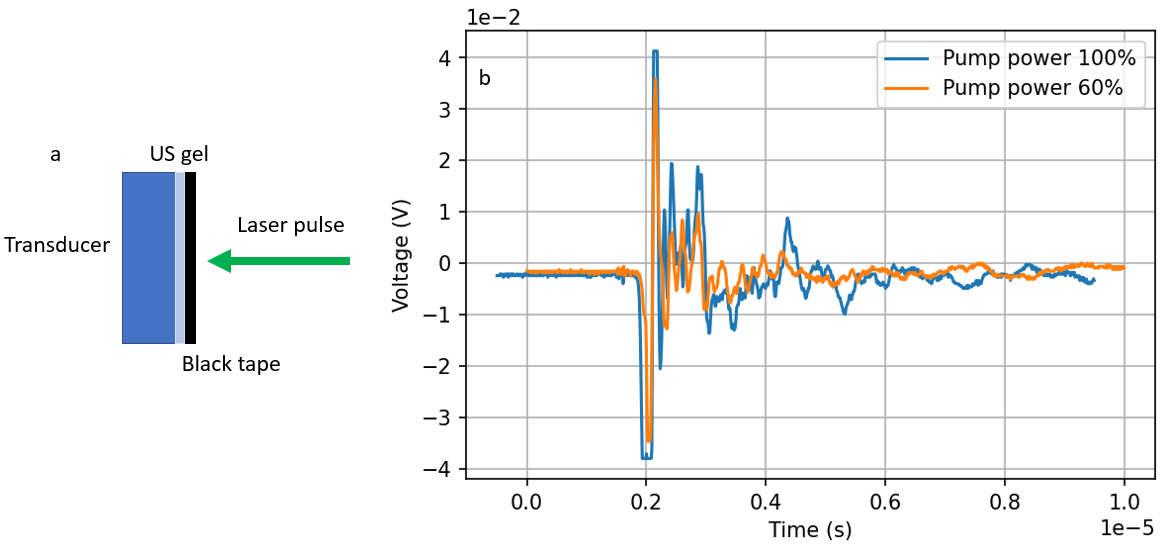
Figure S1. Test experiment. (a). Schematic sketch for measurement~~s~~ of the PA effect in the black tape, (b). Voltage changes during the time when the pump laser pulse illuminates the black tape, blue – power of the pump laser 100%, orange – 60%.


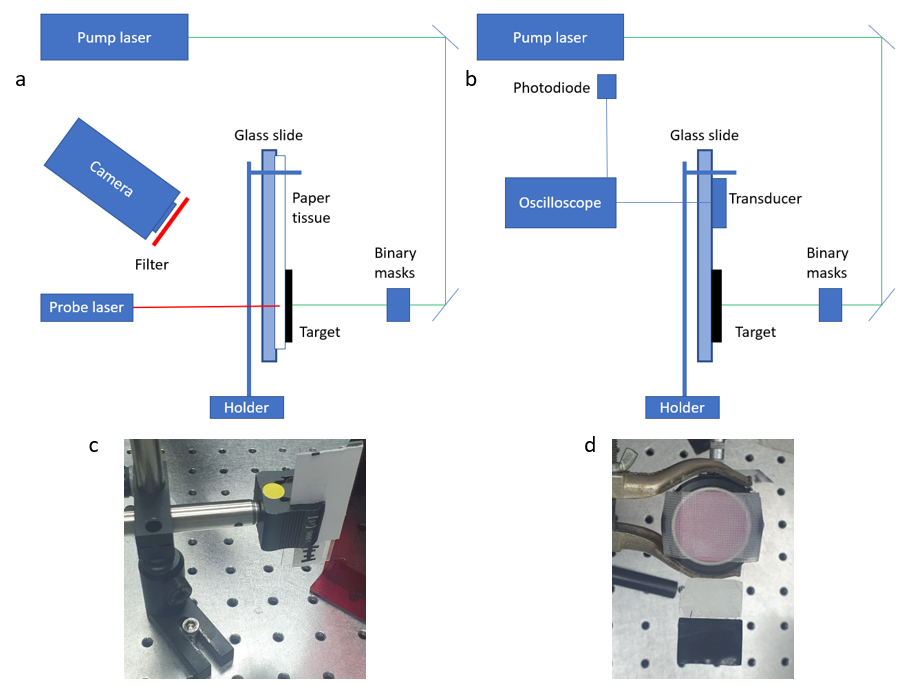


Figure S2. Experimental configurations. (a). Experimental setup for optical measurements with the camera, (b). Acoustic measurements with transducer. (c). Holder for a transducer, (d). Holder for PA targets used for the optical measurements.


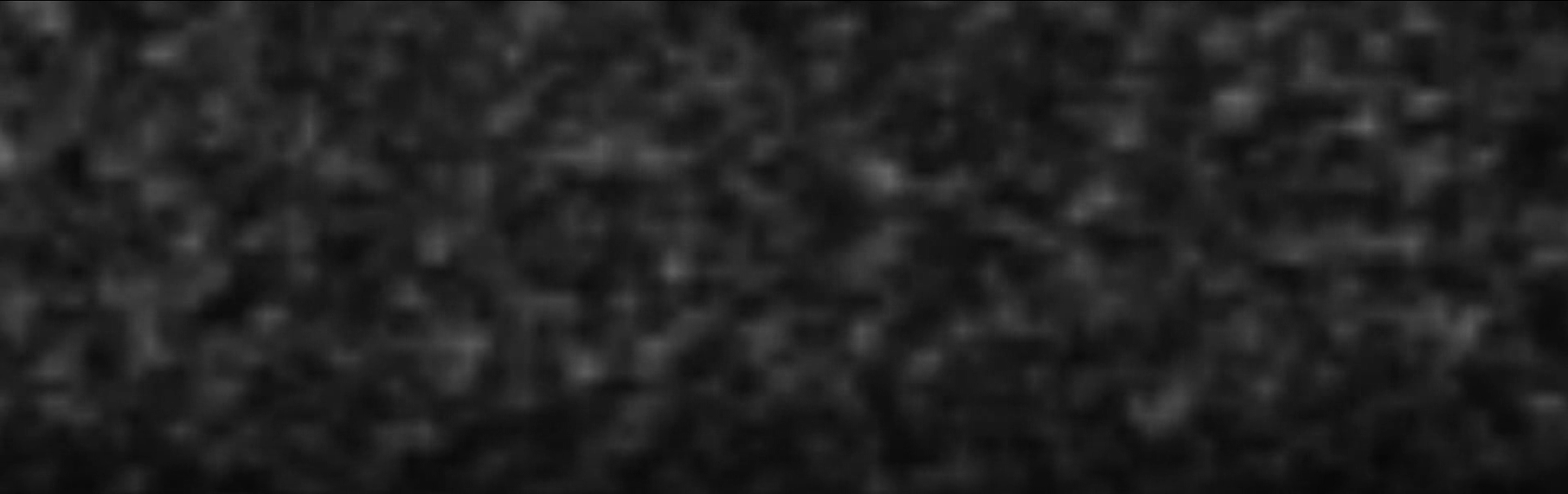


Figure S3. Example of a speckle pattern.


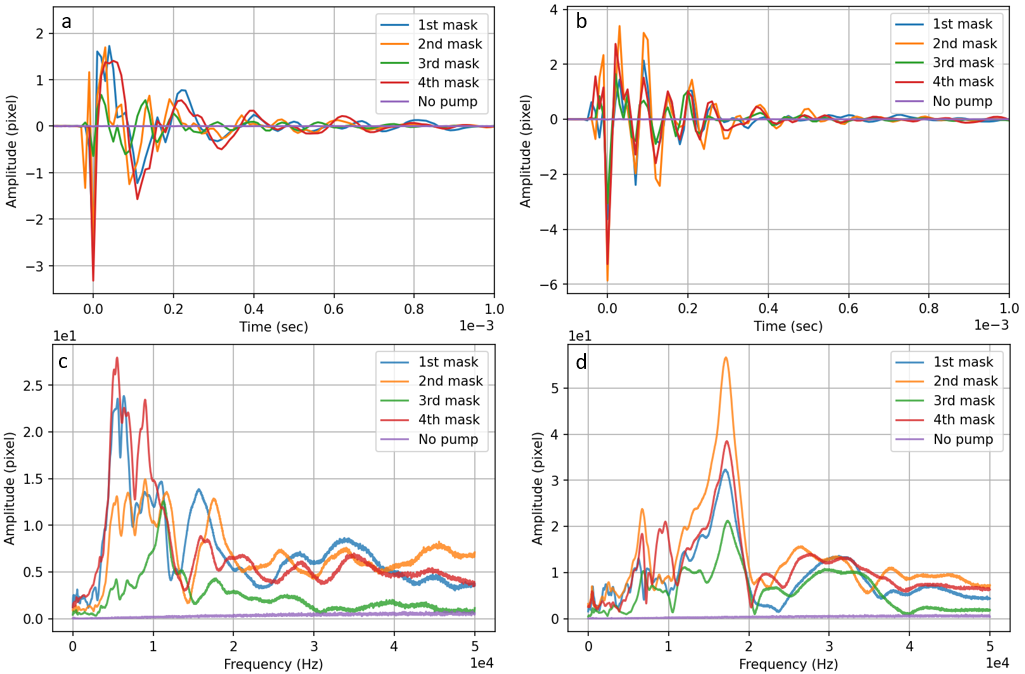


Figure S4. Experimental results. (a) and (b) – Temporal signal for the speckle correlation peak movement along the X and Y axes, respectively, (c) and (d) – Frequency of the speckle correlation peak movement along X and Y axes, respectively.


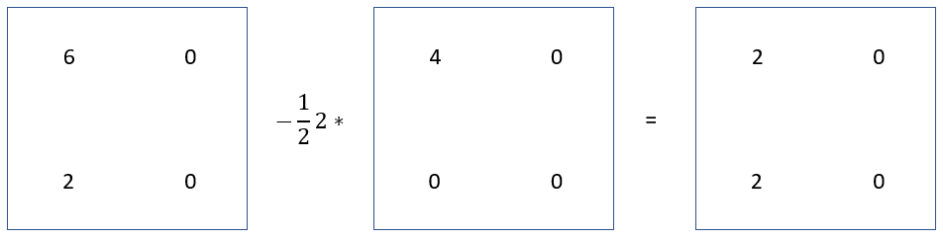
To provide information on the stability of the used approach we have performed theoretical reconstruction, where instead of the ideal theoretical values in the transformed image noise values were used (Figure S5)


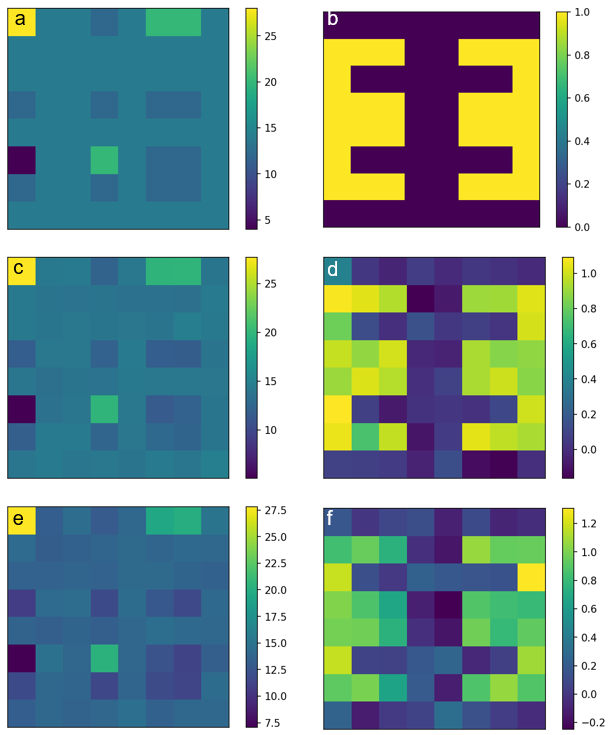


Figure S5. Theoretical reconstruction for noisy images. (a) and (b) –Walsh-Hadamard transformed image of the “EƎ” target and its inverse transformation – reconstruction. (c) and (d) transformed image of the “EƎ” target with ~2.23% relative error added to every element and its reconstruction. (e) and (f) transformed image of the “EƎ” target with ~3.91% relative error added to every element and its reconstruction.

To investigate how measurement error will affect reconstruction we construct following parameter - Image Correlation:

$Image Correlation =1-mean(\left| \hat{s}\left( x \right)-s\left( x \right) \right|)$


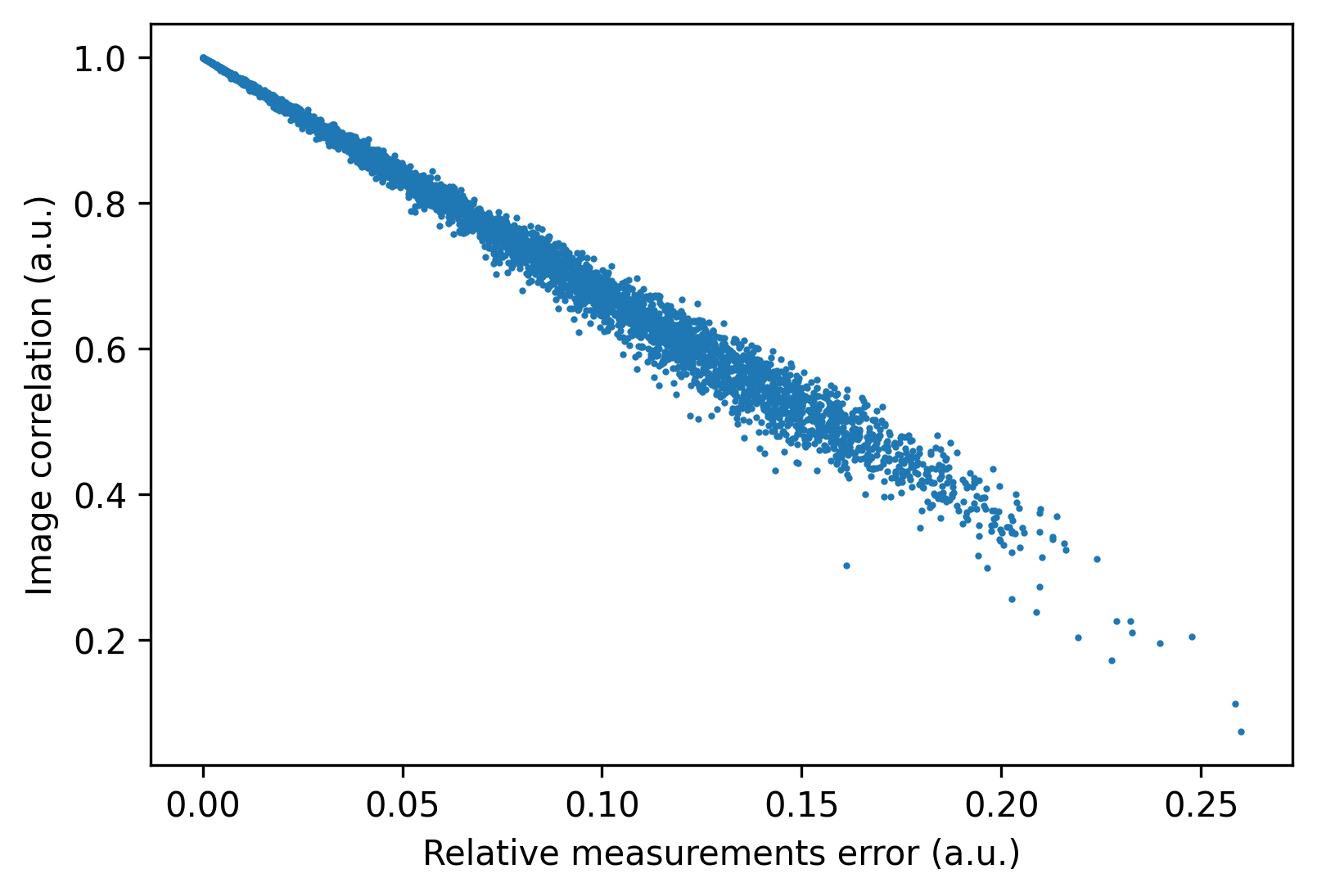


Figure S6. Image correlation for different input errors. Calculated for the “EƎ” target.

Calculations show that ~10% relative error results in undistinguishable “EƎ” pattern in reconstruction image. When only 5 measurement are used for reconstruction, 4 artefacts are observed in corner pixels (Figure S5.a), furthermore higher susceptibility of image correlation on relative error is also observed, when reduced number of elements are used for reconstruction (Figure S5.b)


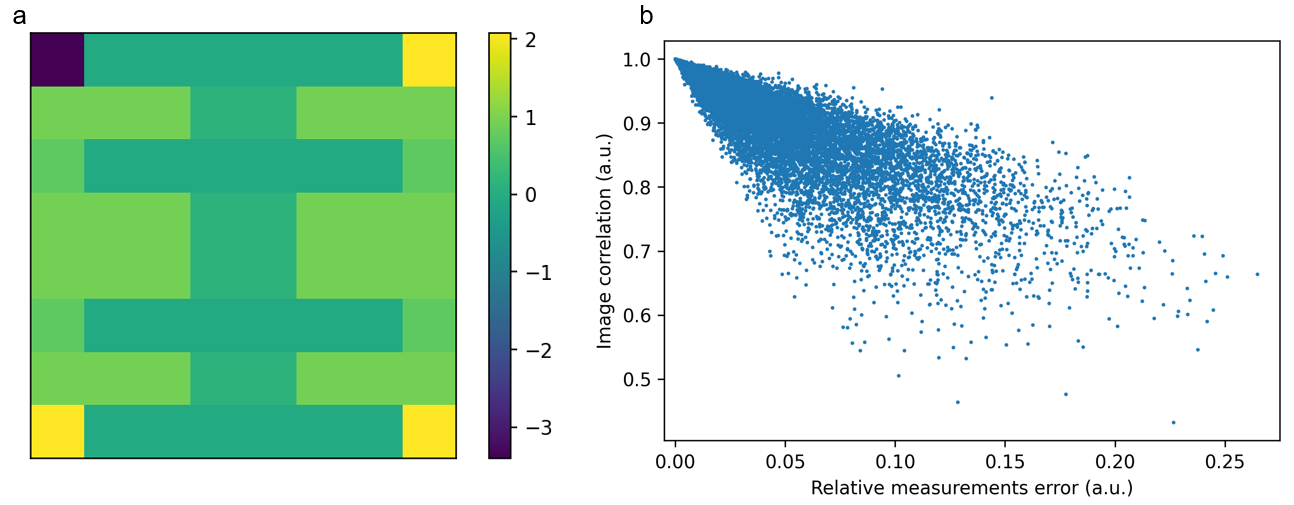


Figure S7. (a) – Reconstruction of the noisy “EƎ” target image, when only 5 unique elements are used. (b) Image correlation for different input errors. Calculated for the “EƎ” target.
